# Automated removal of gradient-induced voltages from 12-lead ECG traces during high-gradient duty-cycle MRI sequences

**DOI:** 10.1186/1532-429X-18-S1-W4

**Published:** 2016-01-27

**Authors:** Mikayel Dabaghyan, Shelley H Zhang, Jay Ward, Raymond Y Kwong, William G Stevenson, Ronald D Watkins, Zion T Tse, Ehud J Schmidt

**Affiliations:** 1 R&D, Mirtech Inc, Boston, MA USA; 2 Radiology, Brigham and Women's Hospital, Boston, MA USA; 3 E-TROLZ Inc, North Andover, MA USA; 4 Brigham and Women's Hospital, Boston, MA USA; 5 Stanford University, Stanford, CA USA; 6 Engineering, University of Georgia, Athens, GA USA

## Background

During cardiac MR imaging (CMR), ECG traces (<10 mV) are highly distorted due to strong gradient-induced voltages (GIV). GIV reaches 1000-3000 mV in high gradient-duty-cycle [GDC = (Total Gradient Activity Time per cardiac cycle)/(Cardiac cycle time)] sequences, for ECG electrodes located farther from magnet's iso-center. An approach which predicted and removed GIV overlaid on ECG traces acquired during CMR using a 19-parameter analytical equation based on Maxwell's equations [Bowtell, MRM. ‘00] and the concomitant fields equation [Bernstein, MRM. '89], was previously verified in 10 subjects at rest and following stress [Zhang et Al, DOI: 10.1002/mrm.25810]. Automating this method would enable its use for efficiently synchronizing CMR (not missing QRS peaks), and for detecting cardiac events (VF, VT) during higher-risk CMR and MR-guided interventions.

To this end we created a self-triggered software pipeline, which requires minimal human intervention, in order to compute and display 12-lead ECG traces without GIV. We verify its efficiency in human subjects during high GDC sequences; SSFP and Short-TR GRE.

## Methods

CMR was performed at 3T (Skyra, Siemens). All data used in the analysis were recoded using a National Instruments Data Acquisition card controlled by MATLAB [Natick, MA]. A commercial CardioLab system (GE, Waukesha, WI) was used to amplify and filter the signal from the ECG leads.

**Figure 1:**
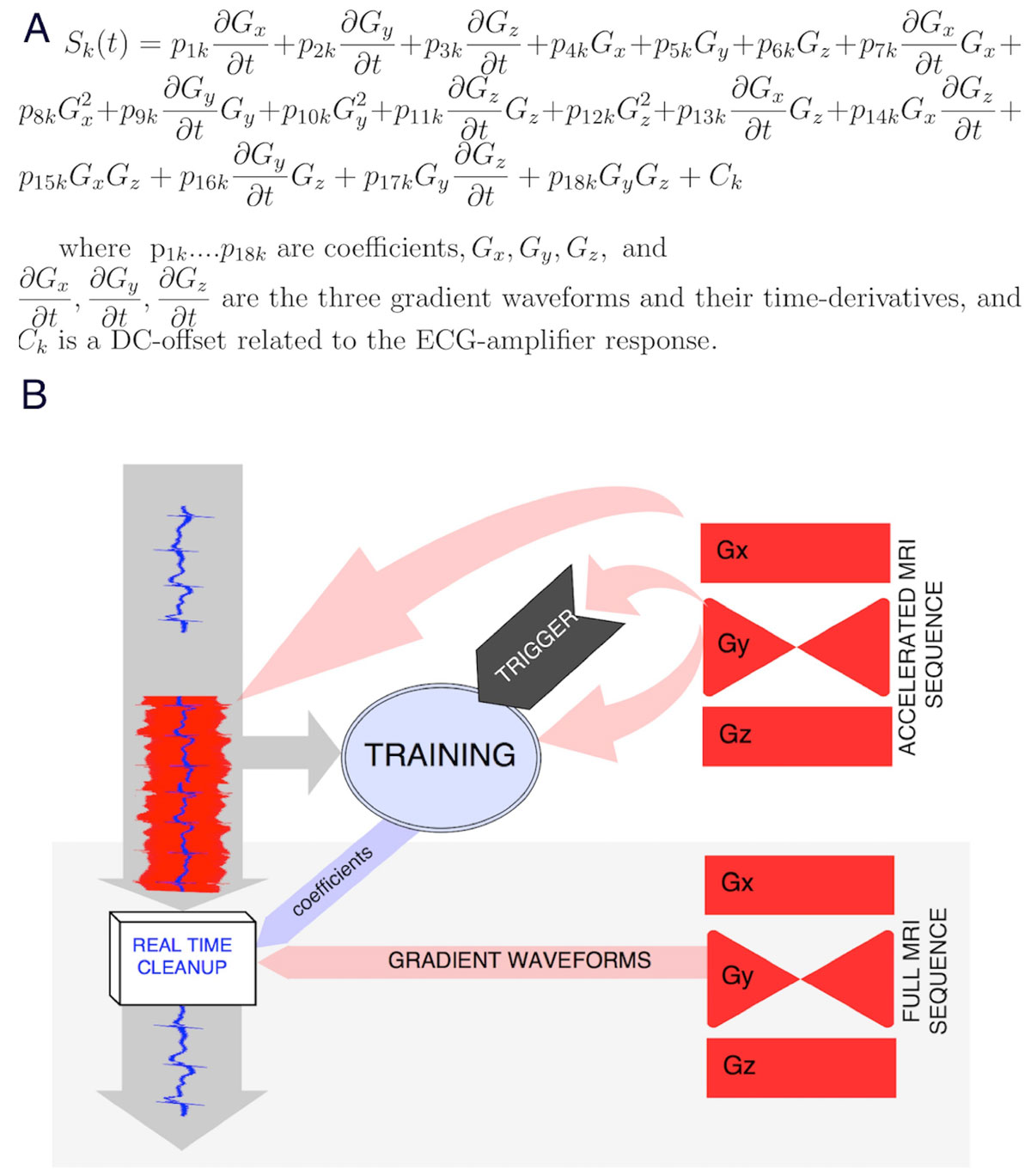
**A) The 19-parameter equation predicting Gradient-Induced Voltages (GIV) using the gradient waveforms**. B) Block-diagram showing the workflow during the automated clean-up routine. A clean portion of the ECG is first acquired and used to calculate a clean ECG template. The program then waits for the trigger to start acquiring the GIV-corrupted ECG signals during the MR scan along with the gradient waveforms. The fitting routine then subtracts the ECG template to obtain the GIV, which are fitted to the equation yielding the coefficients for each channel. These are then used in the real-time ECG monitoring during the subsequent MR scan.

The process is outlined in Figure 1b. Prior to the beginning of imaging, ECG traces without GIV are recorded, after which the software awaits a triggering event to start acquiring data corrupted by GIV. A pulse, marking the start of gradient activity, triggers this acquisition at the start of a training sequence. Imaging gradient-waveforms G_x_, G_y_, G_z_) were simultaneously recorded directly from Siemens hardware. Once all the traces have been acquired for at least three QRS cycles, GIVs are calculated by subtracting the clean ECG template from the corrupted traces, and then fitted to the equation, yielding the coefficients for each sequence. These coefficients are automatically transferred to the real-time program-component, which calculates GIV for each ECG electrode at each time-point during CMR, using the simultaneously-acquired gradient-waveforms, and then subtracts them from the corrupted signals, thereby cleaning the traces (Figure 1).

## Results

**Figure 2:**
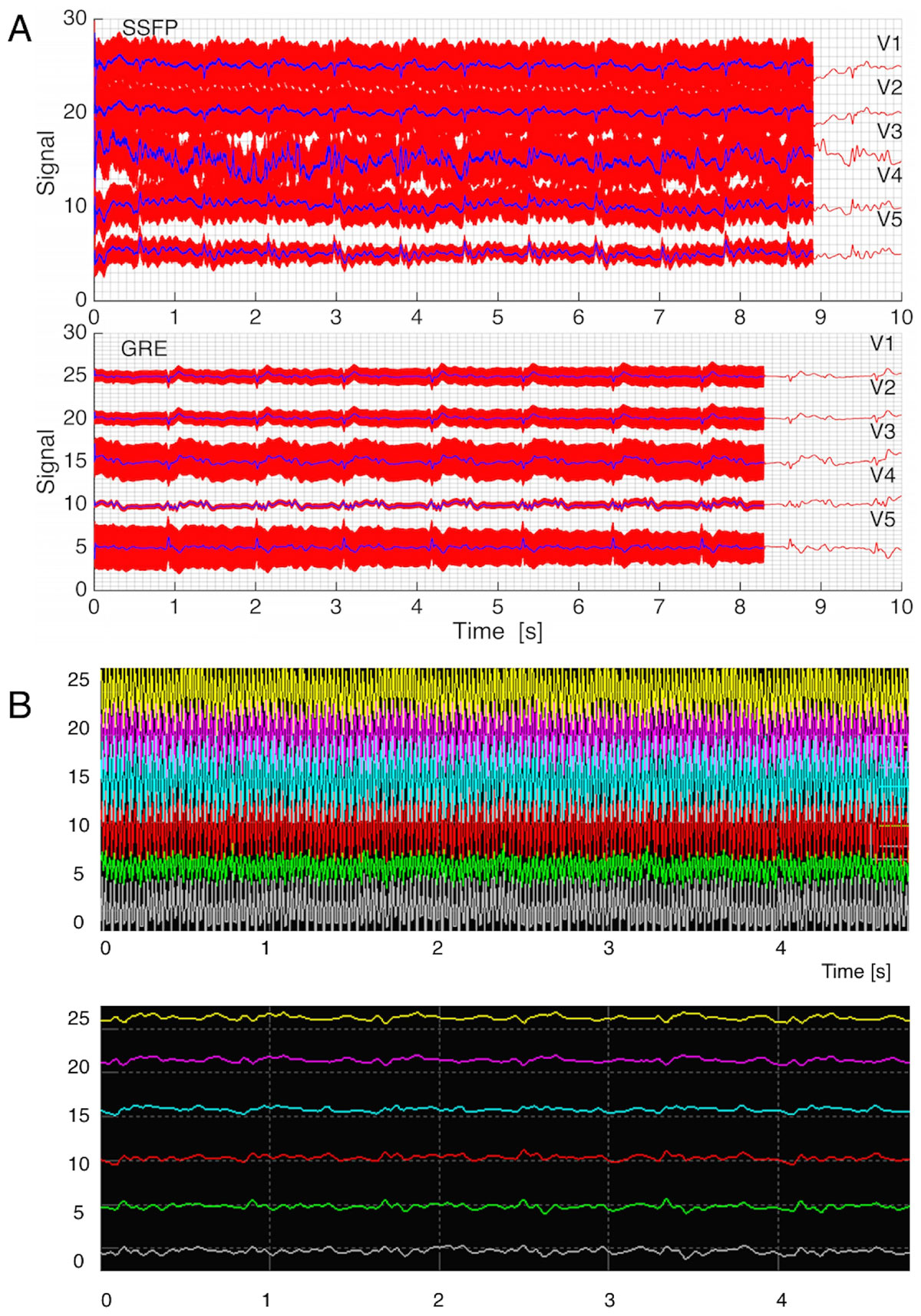
**Sample results of cleaned and GIV-corrupted ECG signals for leads V1-V5 during GRE and SSFP sequences**. A) The measured signal form the ECG leads is plotted in red. One can see the overlaid GIV contribution during the imaging sequence, which disappears at the end of the scan (~9 sec). The cleaned-up ECG data are plotted in blue. B) Screenshot of real-time ECG monitoring during a Gradient-Echo scan following a training routine. The top portion shows the raw signal acquired on the ECG leads during the scan, containing GIV and ECG. The bottom plot shows the real-time output of the clean-up procedure after training.

In two volunteers, we obtained >95% noise removal in 12-lead ECG traces for SSFP and GRE using this automated approach which functioned equivalently to the manual method. The approach succeeded in removing GIV even during changes in heart-rate (Figure 2). Since QRS peaks are clearly discerned in the cleaned ECGs, a VCG-approach [Gregory et al, *DOI: 10.1002/mrm.25078*] provides >90% efficiency in CMR synchronization.

## Conclusions

An efficient automated procedure to remove gradient-induced voltages from ECG traces permits real-time 12-lead ECG-monitoring during high gradient-duty-cycle sequences.

